# Supplementary material for: Decoding bacterial methylomes in four public health-relevant microbial species: nanopore sequencing enables reproducible analysis of DNA modifications
Source: BMC Genomics. 2025 Apr 23;26:394. doi: 10.1186/s12864-025-11592-z (PMC12016153; doi:10.1186/s12864-025-11592-z)
Supplement: Supplementary file 1 — Supplementary Material 1 [file 12864_2025_11592_MOESM1_ESM.pdf]

SUPPLEMENTARY MATERIAL

Decoding bacterial methylomes in four public health-relevant microbial species: Nanopore sequencing enables reproducible analysis of DNA modifications

Valentina Galeone[1,] Johanna Dabernig-Heinz[2], Mara Lohde[3,] Christian Brandt[3,4,] Christian Kohler[5], Gabriel E. Wagner[2], Martin Hölzer[1]

[1]Bioinformatics and Translational Research, Genome Competence Center, Robert Koch Institute, Nordufer 20, 13353 Berlin, Germany.

[2]Diagnostic and Research Institute of Hygiene, Microbiology and Environmental Medicine, Medical University of Graz, Neue Stiftingtalstraße 6, 8010 Graz, Austria.

[3]Institute for Infectious Diseases and Infection Control, Jena University Hospital, Am Klinikum 1, 07747 Jena, Germany.

[4]InfectoGnostics Research Campus, Center for Applied Research, 07743 Jena, Germany

[5]Friedrich Loeffler-Institute of Medical Microbiology, F.-Sauerbruch-Str., 17475 Greifswald, Germany.

SAMPLE ID	Matching SR (260 bp/s)	Matching SR (400 bp/s)
KP04	No	No
KP02	No	Yes
KP13	Yes	Yes
LM46	No	No
LM41	No	Yes
LM54	Yes	Yes
EF35	Yes	Yes
EF26	No	Yes
EF22	No	Yes
SA63	No	Yes
SA67	No	Yes
SA62	Yes	Yes

Table S1) Matching of nanopore sequencing reads (initially sequenced with a translocation speed of 260 bp/s (4 kHz) and later re-sequenced with a translocation speed of 400 bp/s (5 kHz) to Illumina short-read (SR) control data, as reported in the original study (Dabernig-Heinz et al. 2024).

	LAB1				LAB2				LAB3			
SAMPLE ID	Genome size	N50	Contigs	Mean Coverage	Genome size	N50	Contigs	Mean Coverage	Genome size	N50	Contigs	Mean Coverage
KP04	5739683	5416533	3	584	5734002	5410939	3	203	5735503	5412423	3	49
KP02	5463801	5216123	5	272	5423205	5216122	3	173	5463399	5216122	7	56
KP13	6098169	5460880	6*	410	6042480	4826472	9*	134	6100492	5415425	10	99
LM46	3023023	3023023	1	190	3029008	3023019	2*	236	3028999	3023026	2	612
LM41	2999290	2993293	2	175	2999312	2993293	2	275	2999244	2993291	2	647
LM54	3080917	2988921	3	246	3081567	2988919	3	156	2994226	2988921	2	738
EF35	3184789	2978555	6	204	3183881	2978551	6*	190	3172162	2978553	5	54
EF26	3236785	2787038	18	199	3297305	2786914	12	161	3219230	2872321	8	71
EF22	3212785	2783131	10*	265	3238951	2784238	14	160	3242154	2876645	11	31
SA63	2954729	2181338	3	81	2880275	2142915	3	203	2880275	2142917	3*	325
SA67	2821808	2764238	2	75	2791973	2791973	2	224	2791978	2791978	1*	1586
SA62	2884051	847878	7	131	2902075	847790	7*	36	2926242	2891985	3	133

Table S2) Assembly quality metrics and read coverage statistics for analyzed bacterial genomes. The table includes the total size of each genome assembly, N50 values, the number of contigs, and the mean read coverage. Mean coverage values displayed in red indicate isolates with coverage <100X. A contig number with an asterisk indicates the assemblies that were obtained with the flag "--meta" in flye.

**Sample ID**	**Species**	**MicrobeMod *call-methylation***	**Modkit *find-motifs***	**MicrobeMod *annotate-rm* (REBASE)**
KP04	*Klebsiella pneumoniae*	G**AT**C, C**C**W**G**G	G**AT**C, C**C**W**G**G	GATC, CCWGG
KP02	*Klebsiella pneumoniae*	G**AT**C, C**C**W**G**G, G**A**CNNNNNNNG**T**C	G**AT**C, C**C**W**G**G, G**A**CNNNNNNNG**T**C	GATC, CCWGG, GACNNNNNNNGTC
KP13	*Klebsiella pneumoniae*	G**AT**C, C**C**W**G**G, **A**GCNNNNNNC**T**TC	C**C**W**G**G, **A**GCNNNNNNC**T**TC, GAA**A**NNNNNNNG**G**G	GATC, CCWGG, AGCNNNNNNCTTC
LM46	*Listeria monocytogenes*		GAA**GA**C	GAAGAC, CTSAG
LM41	*Listeria monocytogenes*	GA**A**YNNNNNNG**T**C	GA**A**YNNNNNNG**T**C	GAAYNNNNNNGTC
LM54	*Listeria monocytogenes*			
EF35	*Enterococcus faecium*	CAGN**A**C	CAGD**A**C	GATC, CYAANNNNNNNGRTY
EF26	*Enterococcus faecium*	CYA**A**NNNNNNNGR**T**Y	CYA**A**NNNNNNNGR**T**Y	CYAANNNNNNNGRTY
EF22	*Enterococcus faecium*	CYA**A**NNNNNNNGN**T**Y	CYA**A**NNNNNNNGR**T**Y	CYAANNNNNNNGRTY
SA63	*Staphylococcus aureus*	GW**A**GNNNNNNGA**T**, CG**A**NNNNNNNN**T**CC	GW**A**GNNNNNNGA**T,** CG**A**NNNNNNNN**T**CC	GWAGNNNNNNGAT, CGANNNNNNNNTCC
SA67	*Staphylococcus aureus*	GA**A**GNNNNNNT**A**C, CC**A**YNNNNNNNDTY	CC**A**YNNNNNNNR**T**C, CC**A**YNNNNNNN**T**TYG	GAAGNNNNNNTAC, CCAYNNNNNNNTTYG
SA62	*Staphylococcus aureus*	CC**A**YNNNNNNN**T**GT, **A**GGNNNNNNGA**T**	CC**A**YNNNNNNN**T**GT, **A**GGNNNNNNGA**T**	CCAYNNNNNNNTGT, AGGNNNNNNGAT

Table S3) Comparison of methylated motifs identified by MicrobeMod's call-methylation pipeline, Modkit (find-motifs), and the MicrobeMod annotation pipeline.

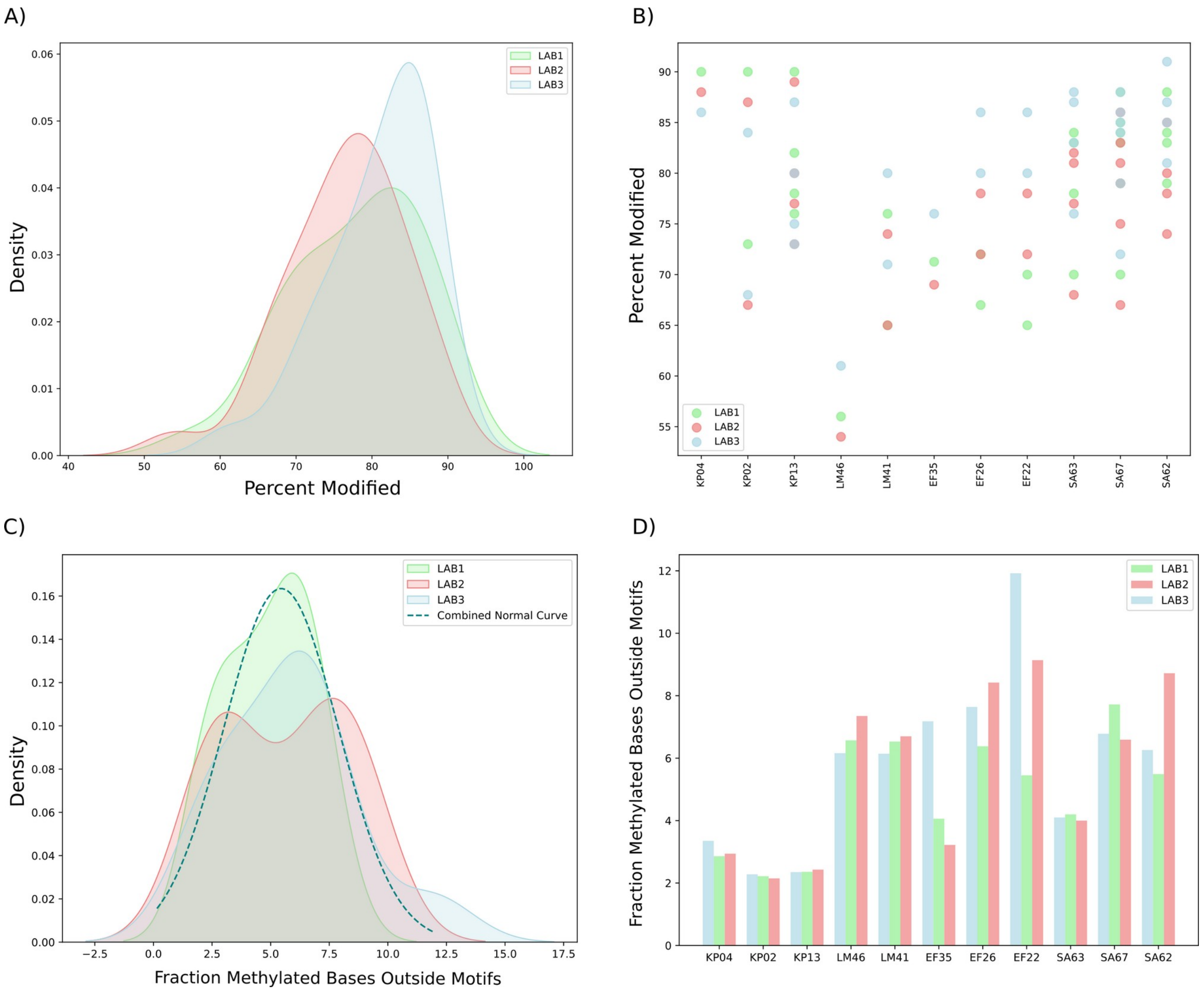

Figure S1) Top panels: Average *Percent Modified* values of methylated bases within detected motifs for 6mA. **(A)** Distribution of *Percent Modified* values for each replicate. **(B)** Distribution of *Percent Modified* values, divided by sample ID. Most motifs were validated from the literature, including the motif in LM46, which shows a lower *Percent Modified* (55-60%). Based on these results, we defined bases with *Percent Modified* > 0.5 as methylated in our study. Bottom panels: Fraction of methylated bases (*Percent Modified* > 0.5) that are not explained by a motif for 6mA. **(C)** Distribution for each replicate. **(D)** Distribution divided by sample ID. These results indicate that approximately 95% of methylated bases are explained by a motif.

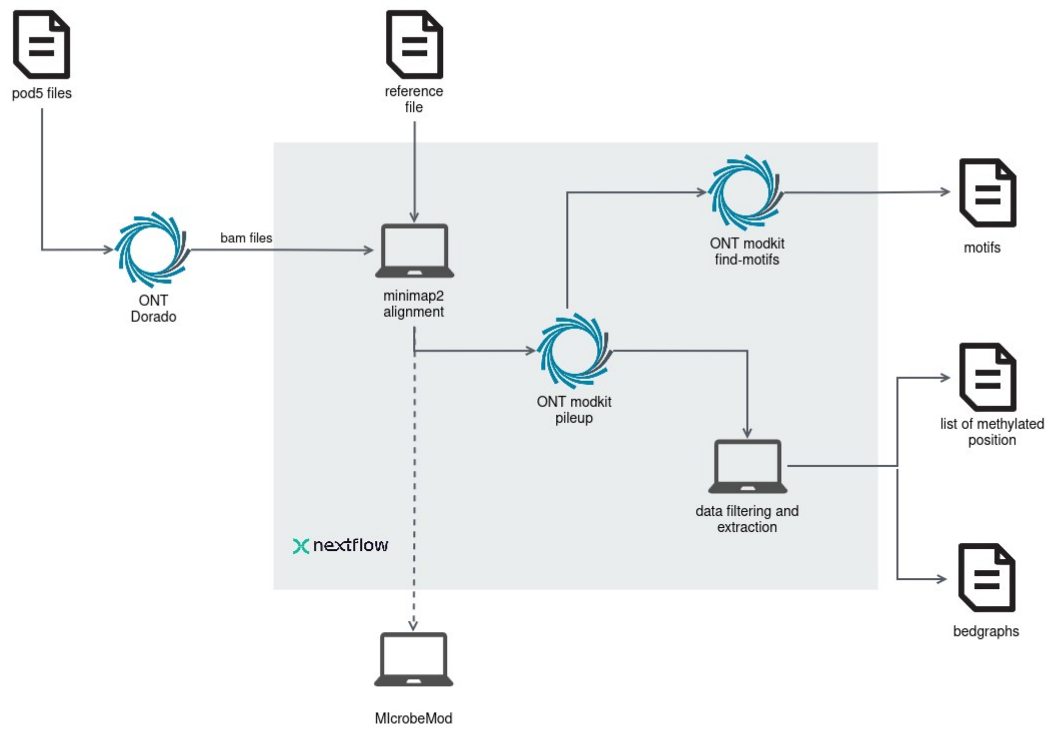

Figure S2) Schematic overview of the in-house pipeline (covering the analysis steps highlighted in the grey box) used for the preprocessing, methylation detection, and motif extraction analysis. The pipeline is publicly available at: https://github.com/rki-mf1/ont-methylation.

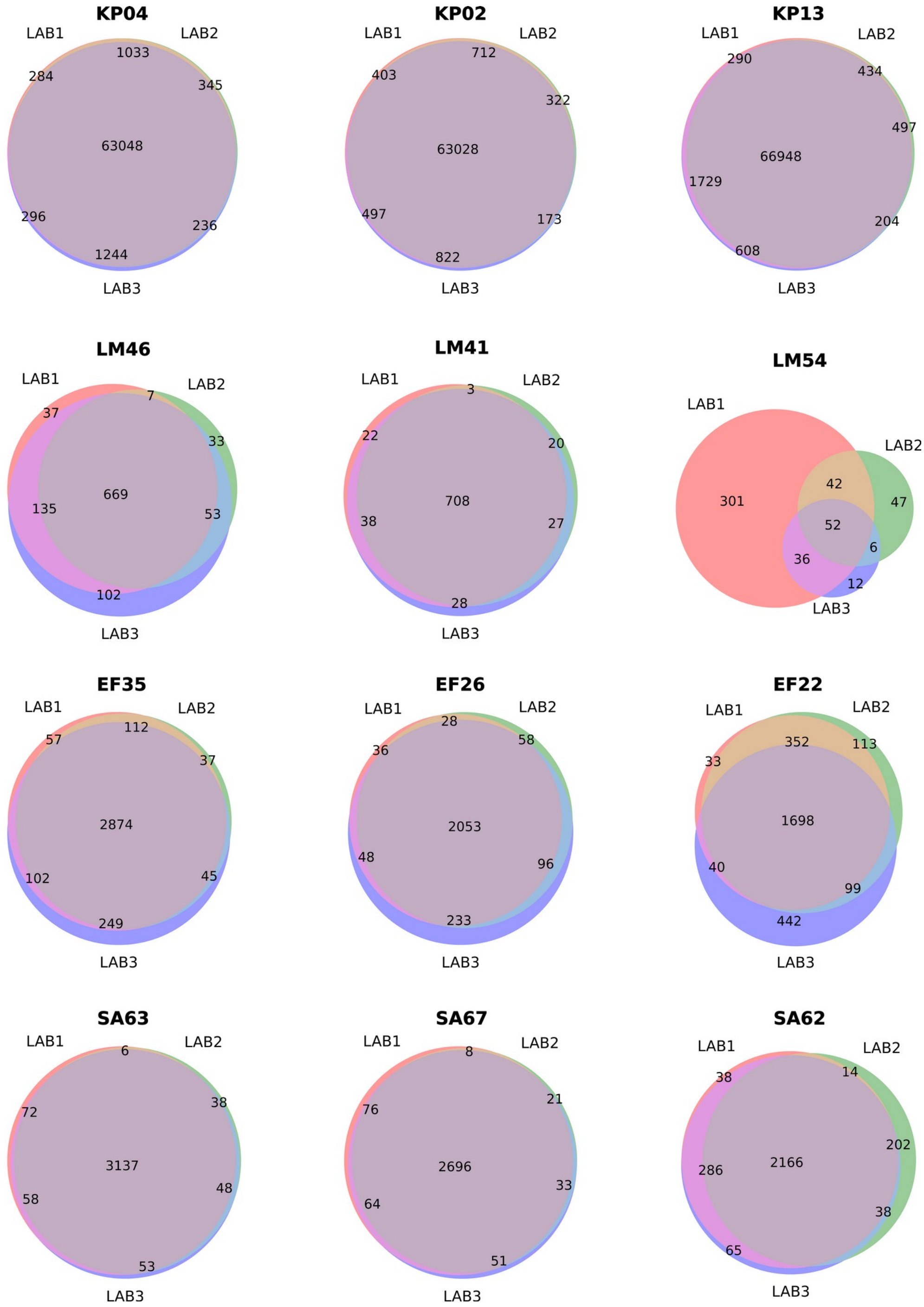

Figure S3) Venn diagram of methylated 6mA positions (*Percent Modified* > 0.5) across replicates. We compared the results of the three labs, using the assemblies obtained in LAB1 as references, to measure whether the same bases were detected as methylated. . Isolate LM54 is the only sample with no detected motifs and exhibits high disagreement among the few detected 6mA bases. Isolates EF22 and SA62 show higher disagreement, likely due to lower sequencing coverage in certain labs. Additionally, in isolate LM46, the GAAGAC motif has a lower average *Percent Modified* (around 0.6), leading to a greater number of bases that are not consistently shared across replicates.

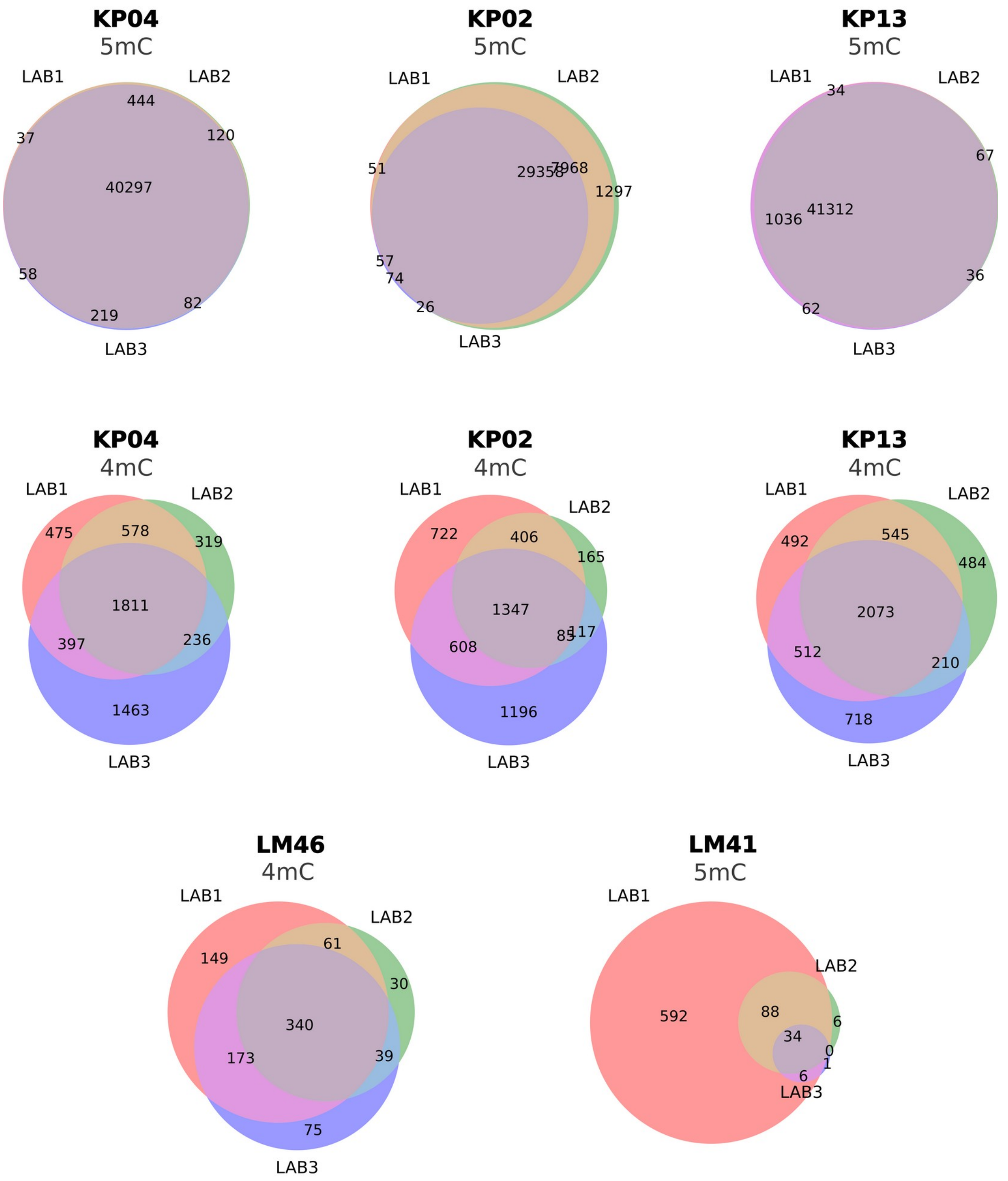

Figure S4) Venn diagram of methylated 5mC and 4mC positions (Percent Modified > 0.5) across replicates. We compared the results of the three labs, using the assemblies obtained in LAB1 as references, to measure whether the same bases were detected as methylated. The results for 5mC in *K. pneumoniae* show a high agreement of methylated positions, with the exception of LAB3 for KP02. In contrast, 4mC bases in *K. pneumoniae* show greater disagreement and no detected motifs. Only isolate LM46 has methylated 4mC in the GAAGAC motif, but its relatively low average Percent Modified (~0.6) leads to imperfect agreement between replicates. Additionally, isolate LM41 contains methylated 5mC bases in the partially methylated TCGA motif.

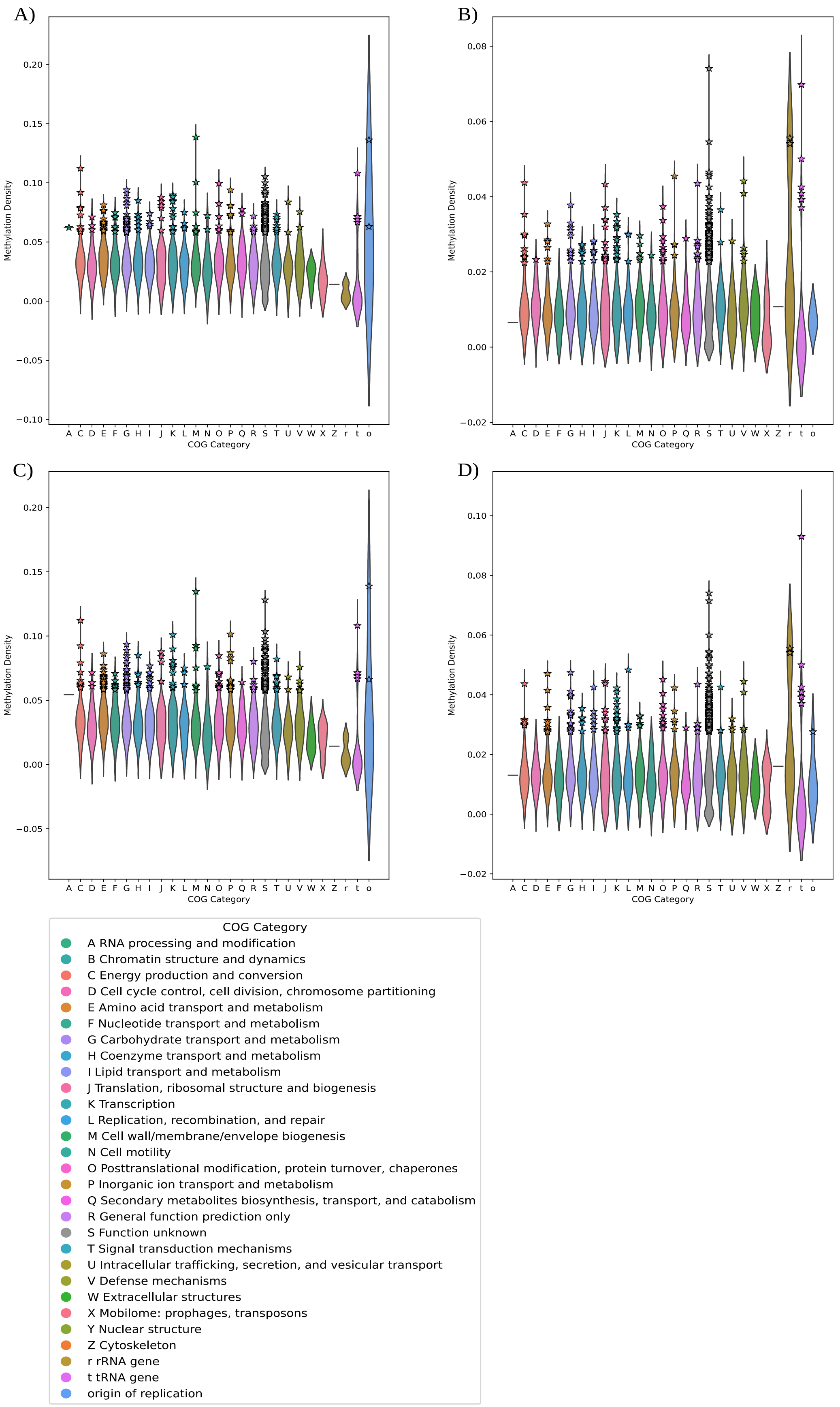

Figure S5) Violin plots of methylation density of the genes of KP02 and KP13 isolates: panels **A)** and **B)** show 6mA and 5mC methylation density for KP02, while panels **C)** and **D)** show 6mA and 5mC for KP13. The genes are grouped by gene function according to the COG classification from Bakta.

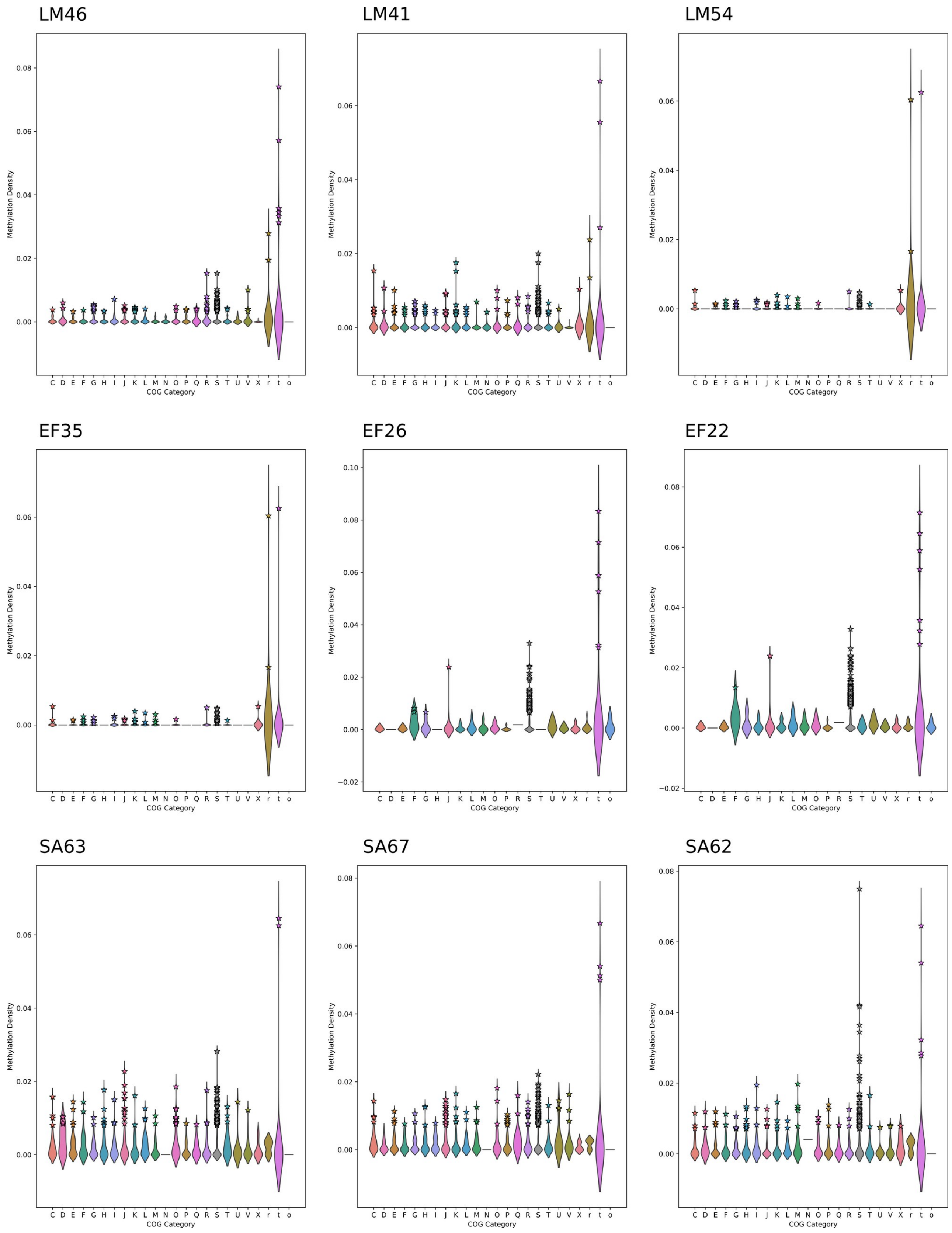

Figure S6) Violin plots of methylation density of the genes of LM, EF, and SA for 6mA. The genes are grouped by gene function according to the COG classification from Bakta (for the color code, please refer to the COG legend in **Supplementary Figure S5**).

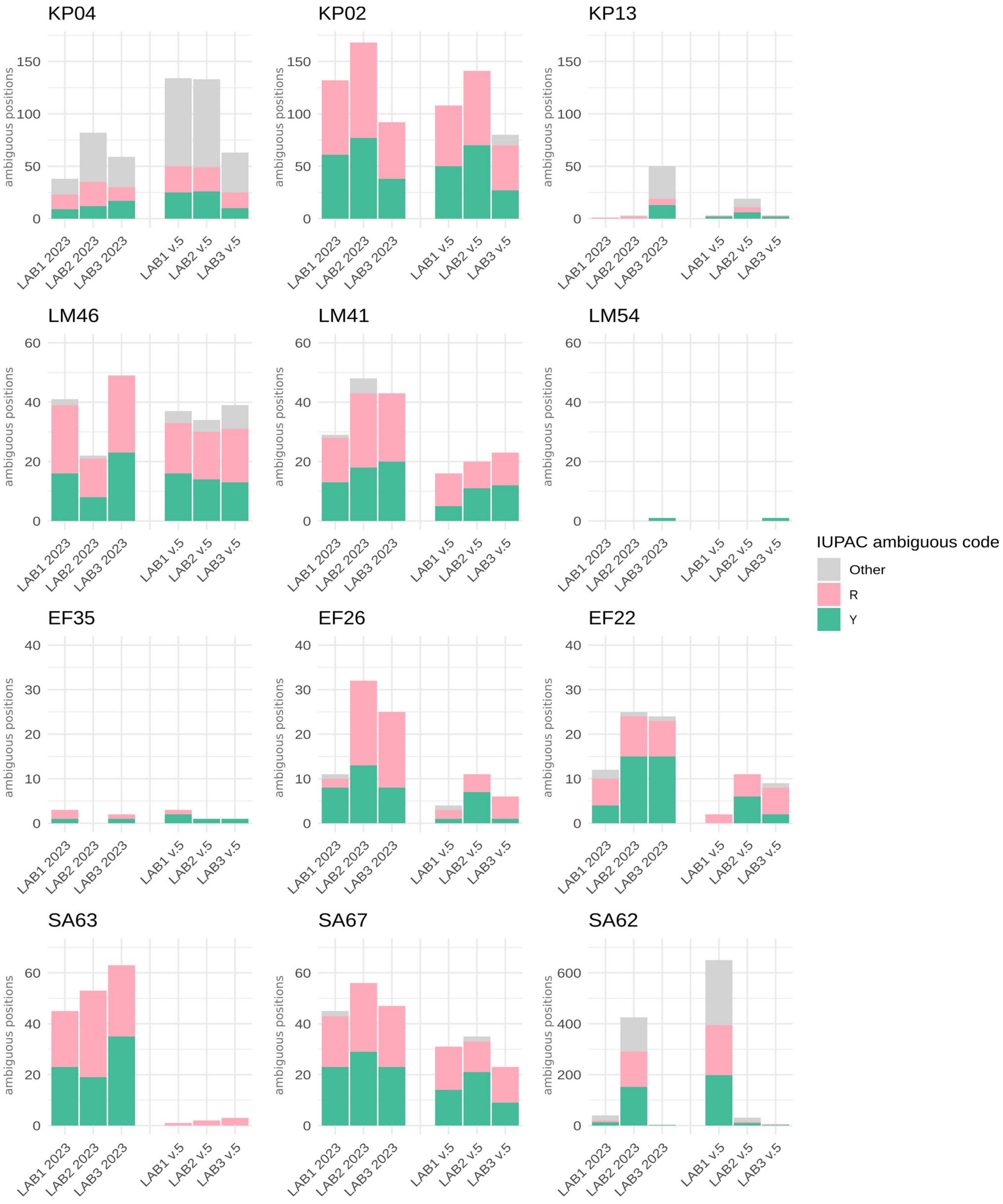

Figure S7) Comparison of ambiguous positions (R = A or G, Y = C or T in IUPAC code) between the old model 2023-09-22_bacterial-methylation (2023) and the new Dorado model v5 across three replicates obtained with the pipeline MPOA. The data show a decrease in the number of ambiguous positions for most samples, with the exception of KP02 and LM46.

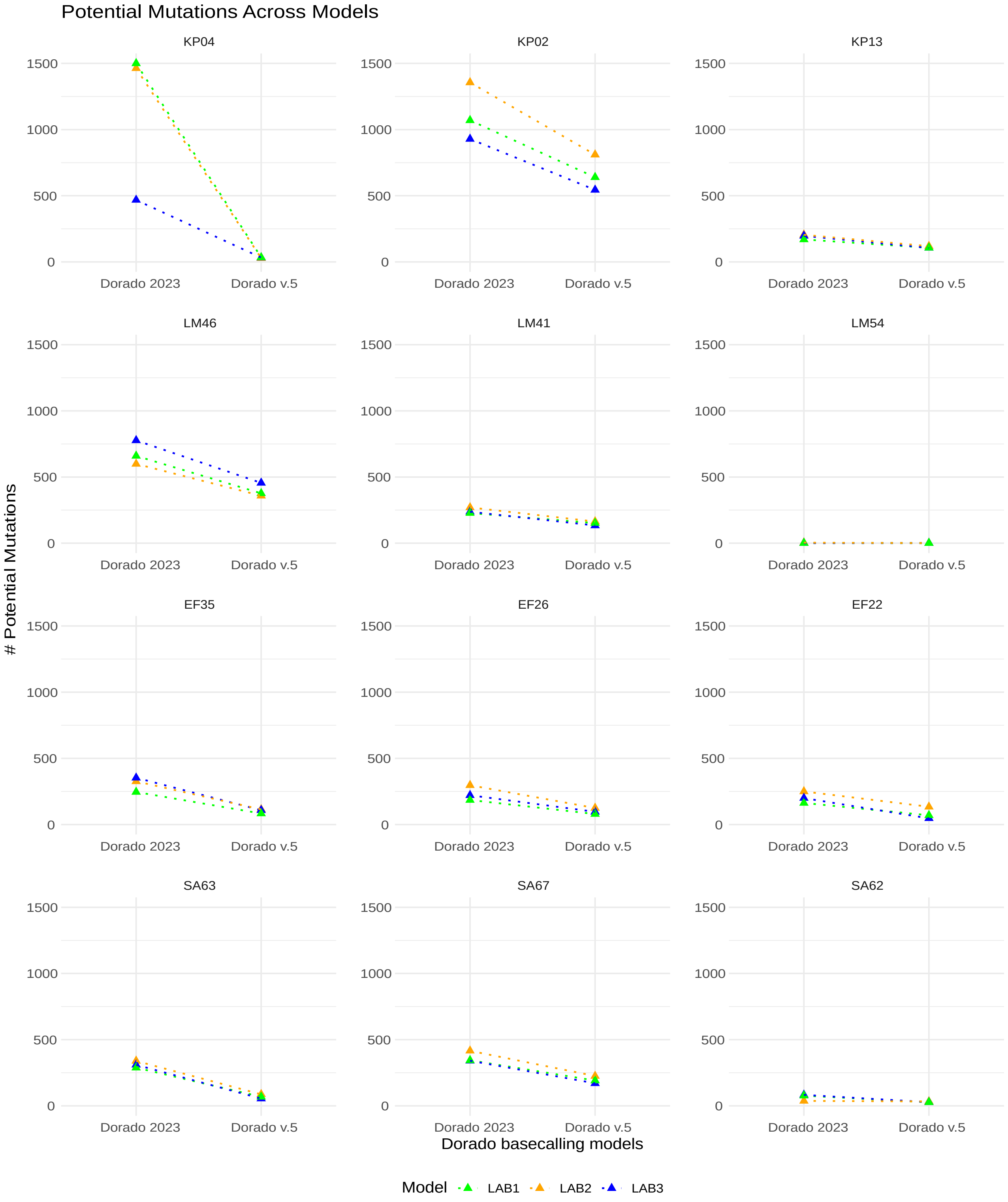

Figure S8) Comparison of the number of potential mutation sites identified by Hammerhead for the old model 2023-09-22_bacterial-methylation (2023) and the new Dorado model v5.

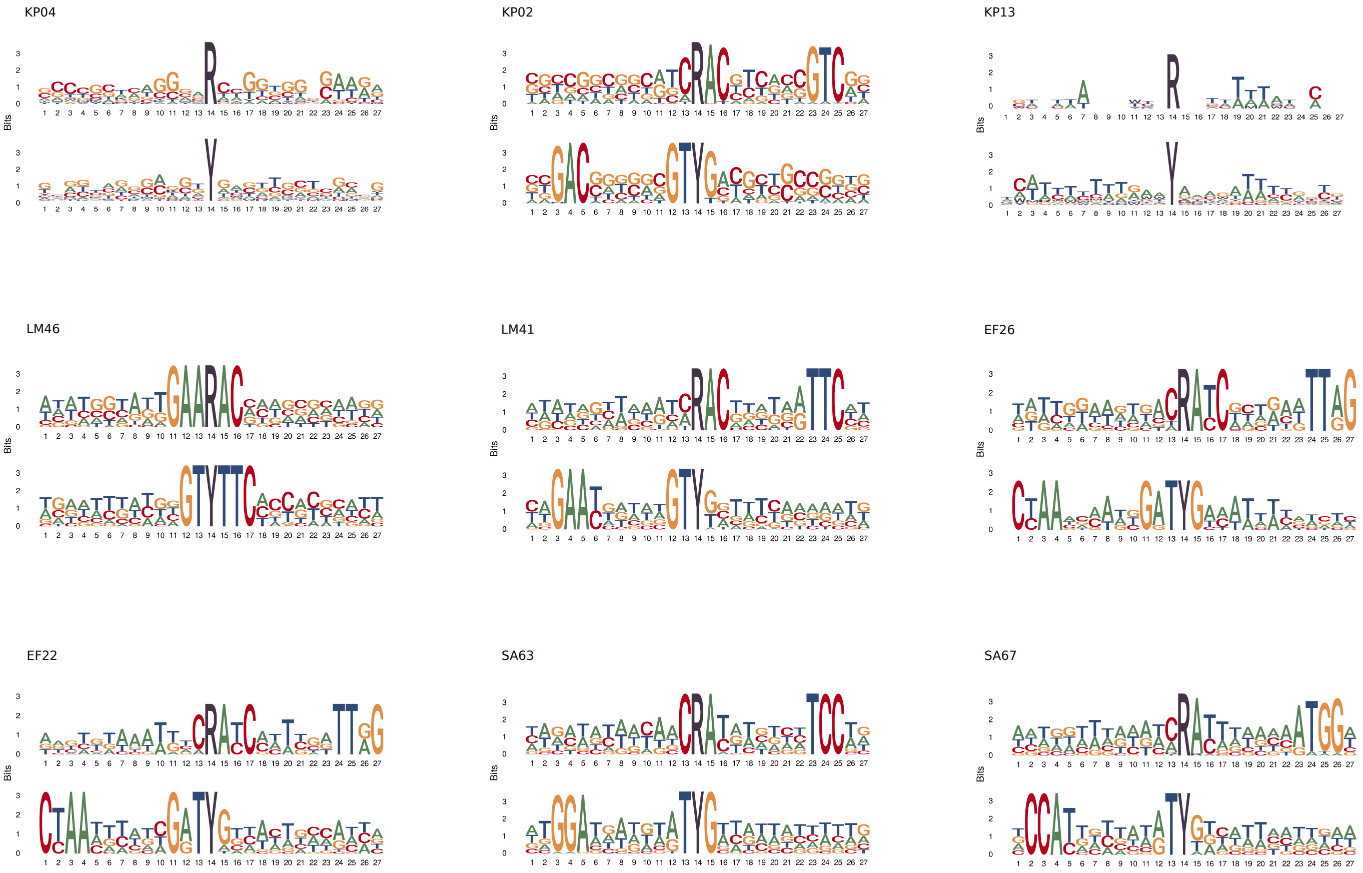

Figure S9) DNA logos generated with MPOA for the strains with R and Y ambiguous positions, using the Dorado basecalling models 2023-09-22_bacterial-methylation on the LAB3 dataset, highlighting the bases surrounding ambiguous positions. In most cases, motifs detected with our pipelines/MicrobeMod are clearly identifiable within the sequences.
